# Parkinson’s disease patients benefit from bicycling - a systematic review and meta-analysis

**DOI:** 10.1038/s41531-021-00222-6

**Published:** 2021-09-24

**Authors:** Marianne Tiihonen, Britta U. Westner, Markus Butz, Sarang S. Dalal

**Affiliations:** 1 Institute of Clinical Neuroscience and Medical Psychology, Medical Faculty, Heinrich Heine University Düsseldorf, Düsseldorf, Germany; 2 Center for Music in the Brain, Department of Clinical Medicine, Aarhus University & The Royal Academy of Music Aarhus/Aalborg, Aarhus, Denmark; 3 Center of Functionally Integrative Neuroscience, Department of Clinical Medicine, Aarhus University, Aarhus, Denmark; 4 Radboud University Nijmegen, Donders Institute for Brain, Cognition and Behaviour, Nijmegen, The Netherlands

**Keywords:** Parkinson's disease, Rehabilitation, Parkinson's disease, Outcomes research, Quality of life

## Abstract

Many Parkinson’s disease (PD) patients are able to ride a bicycle despite being severely compromised by gait disturbances up to freezing of gait. This review [PROSPERO CRD 42019137386] aimed to find out, which PD-related symptoms improve from bicycling, and which type of bicycling exercise would be most beneficial. Following a systematic database literature search, peer-reviewed studies with randomized control trials (RCT) and with non-randomized trials (NRCT) investigating the interventional effects of bicycling on PD patients were included. A quality analysis addressing reporting, design and possible bias of the studies, as well as a publication bias test was done. Out of 202 references, 22 eligible studies with 505 patients were analysed. An inverse variance-based analysis revealed that primary measures, defined as motor outcomes, benefitted from bicycling significantly more than cognitive measures. Additionally, secondary measures of balance, walking speed and capacity, and the PDQ-39 ratings improved with bicycling. The interventions varied in durations, intensities and target cadences. Conclusively, bicycling is particularly beneficial for the motor performance of PD patients, improving crucial features of gait. Furthermore, our findings suggest that bicycling improves the overall quality-of-life of PD patients.

## Introduction

Goal-directed physical exercise and general physical activity have been demonstrated to alleviate both motor and cognitive symptoms in Parkinson’s disease (PD) in addition to the standard pharmaceutical and surgical treatments^1,2^. Among the diversity of physical exercise forms, the need to develop a goal-based rehabilitation has been highlighted^1^, as thus far there has not been sufficient knowledge to target or customize adjuvant forms of exercise to patient’s individual needs^3^. Therefore, a more thorough understanding of the efficacy of different forms of exercises such as bicycling is important, as new forms and technologies around exercise therapies are being increasingly established^4–6^.

Exercise-based training in particular can be targeted to enhance functional mobility by utilizing enhanced strength, endurance, balance and flexibility to support efficient performance of specific tasks^3^. While there is no conclusive evidence that exercise would terminate disease progression, it can be considered as disease-modifying when underlying pathological or pathophysiological disease processes are delayed and being accompanied by improvement in clinical signs and symptoms^3^. In addition to studies with patients, work on animal models indicate that physical activity can have neuroprotective effects on the brain by enhancing neuroplasticity and reinforce structural and morphological changes leading to the attenuation of age-related cognitive decline^7^.

In 2010 it was reported that some individuals diagnosed with PD, while indicating severe freezing of gait (FOG), were nevertheless able to ride a bicycle easily^8,9^. Since then, the ability to preserve the skill to ride a bicycle, while otherwise being severely limited by the symptoms of PD, has been shown to positively influence cardiovascular fitness, motor skills, overall coping, feeling of independency, social inclusion and cognitive skills^10–13^.

To investigate the current state of the art, we conducted a meta-analysis with a review on the literature on bicycling as an adjuvant form of exercise for PD. The goal of this review is to provide a characterization of the status of bicycling exercise regimen for PD patients and to identify features that need further research. In reference to the Patient, Intervention, Comparison, Outcome framework (PICO)^14^, this review aims to quantify if PD patients (P), benefit from bicycling intervention (I) compared with pre- and post-measures of the same population, or compared with the outcomes an alternative exercise intervention, or standard treatment given to another PD group (C). The relevant outcomes are measured as changes in physical and cognitive measures, and in quality-of-life (O).

## Methods

### Eligibility criteria

The review was pre-registered in the International Prospective Register of Systematic Reviews (PROSPERO) [CRD42019137386]. Only original English and German language peer-reviewed research articles were included. The criteria for eligible studies were as follows: only original reports applying quantitative measures to investigate the effects of an intervention on patients diagnosed with PD, providing quantitative pre- and post-outcome measures, with the appropriate measures of averages and variability, on the efficacy of the treatment. Regarding the definition of bicycle exercise, studies with recumbent, tandem, motorized, non-motorized and stationary bicycle ergometers were included. Furthermore, studies were also included if the pedalling was done with hands instead of feet. Studies were not eligible if the intervention was only imagined, or only performed using virtual reality without an actual ergometer. No limitations were set with respect to the control treatment, as long as the treatment did not involve bicycling.

Reviews and meta-analyses were excluded. Furthermore, studies were excluded if their primary outcomes measured neurophysiological or metabolic activity. While randomized control trials (RCT) have been the gold standard of empirical testing^15^, and the preferred study design to be included in a meta-analysis, also non-randomized trials (NRCT) were included here. The inclusion of NRCT studies was considered justified as they can be the main source of evidence for several intended effects of interventions^16^. Including NRCT studies is increasingly common and encouraged in particular among non-pharmacological studies, investigating the effectiveness of therapeutic interventions^16–19^.

### Search strategy

The PubMed database for biomedical literature was searched for studies published between January 2010 and February 2020. This was set as the time frame, as the observation of preserved bicycling ability in a PD-patient with severe FOG was first reported 2010^8^. The keywords ‘bicycl*’, ‘cycling; bik*’ and ‘Parkinson, bicycl*’ as a MeSH term were used.

### Study selection and data extraction

The study screening was done independently by two reviewers, M.T. and B.U.W., using the Covidence Systematic review software^20^. First, the imported articles were screened based on title and abstract, then based on the full text. Any occurring conflicts on inclusion were solved by the third reviewer, S.S.D. Upon inclusion, the qualitative and quantitative information about each study was extracted into three different tables:

- Publication: Authors, publishing year
- Study: Study design, number of individuals in treatment and control group
- Effect size measures: Quantitative measures on pre- and post-treatment
- Participant demographics: Age, gender, disease duration, medication
- Intervention characteristics: Bicycle type, cadence (the number of pedal strokes in a minute, usually measured as rounds per minute, RPM), treatment session duration, overall treatment duration, exercise intensity in heart rate and perceived exertion.

### Meta-analysis

#### Quality analysis

A versatile checklist developed for evaluating primary research papers, the Standard Quality Assessment Criteria tool (QualSyst), was used to estimate the quality of the included studies^21^. The QualSyst tool was deemed appropriate as it is developed in particular for meta-analyses including both RCT and NRCT studies, addressing the overall quality of the studies with a 14-item checklist concerning the internal validity of the studies, possible bias, as well as the quality of the reporting. The reviewers M.T. and B.U.W. assessed the included studies independently, answering each checklist question with Yes, Partial, No or Not applicable. This enabled the computation of a score through the QualSyst tool, assessing the overall quality of a study. The quality assessment was not used to set cut-offs for study inclusion, rather it was used as additional information about the overall quality of the included studies. The final score is based on the average of the score from both independent reviewers. A two-sample *F*-test for the variance of the scores as well as a paired *t*-test to test for a difference in the ratings given by the reviewers were conducted.

#### Publication bias

To address whether the included literature might have been subject to publication bias, the small sample bias method was applied for the primary outcome measures to test for the presence of a possible bias^22^. The method is based on the assumption that studies with high effect sizes will most probably get published, while studies with low effect sizes will not^23^. The risk of non-significant and small effect sizes is particularly high for studies with small sample sizes. This would mean that the sample of the included studies could show a lack of small studies featuring very small or negative effect sizes, while still including small studies with larger effect sizes and stronger statistical significances^24^.

#### Effect size of the treatment

The analysis of the treatment efficacy was based on the generic inverse-variance method which uses the effect size, and a weighted measure of variance for each study to calculate the pooled effect size describing the overall effect. The given weight for each study is the inverse of the variance. Here, the effect sizes were based on continuous outcome data, and they were pooled using a random-effects model^24^, assuming that not all studies come from the same population. The Hedges’ bias-corrected standardized mean difference (*g*) was used to calculate the effect size. To calculate the between-study variance, tau², the Sidik-Jonkman method (SJ) was chosen.

The analysis was conducted with the R^25^ and RStudio software^26^, by using the meta^22^ and metaphor^27^ packages which were developed for meta-analyses. For the (R)CT studies, the individual effect size and the variance were calculated for the post-measures of the treatment and the control group. Pseudo-randomized studies, and studies that applied the same inclusion criteria for the treatment and control group of PD patients were included in the (R)CT-group, thus, the R in the acronym RCT is in parentheses. The rest of the studies consisted of two types of studies; some compared PD patients with healthy participants, and some applied a repeated design, comparing PD patients before and after treatment. As the included studies already would include repeated measure designs with only PD patients, in which the individual effect size would be calculated for the pre- and post-treatment measures, the outcomes of the rest of the studies were grouped together and analysed in the same manner, and thus are called repeated trials (RT). This means that the outcomes of the healthy participants were excluded, and only the individual effect sizes of the PD’s pre- and post-treatment measures were compared. Ideally, an analysis comparing healthy participants and PD patients would be based on the comparison of difference measures of both groups. Nevertheless, this approach was not deemed feasible as a reliable calculation of the variance of each participant would have required an access to the individual data of each participant.

In case of studies with multiple measuring time points, the earliest and the latest time points were chosen. Also, in the case of studies which had more than two treatment options, cycling was contrasted, if available, with no-treatment or with standard care. In cases where the required information was not reported explicitly enough, the authors were contacted.

#### Primary and secondary measures

In the initial analysis, all included studies were grouped, and the effect size of the primary measures was tested. A primary measure was defined as it was stated in the corresponding original paper. If there were multiple primary measures or if a primary measure was not named explicitly, a measure was chosen for the analysis that was best aligned with the rest of the outcome measures of the meta-analysis. Next to investigating the primary measures, secondary outcomes were also analysed. First, outcome measures that could be defined to be functional were tested for their effect size. An outcome was defined as ‘functional’ if it could be considered to enable general movement and mobility of the body. Thereafter, the secondary outcomes were investigated in more detail, as it was investigated whether bicycling influenced four outcomes of gait (Cadence, Step length, Speed) and walking capacity (6 min walking test, 6MWT), as well as Bradykinesia, Tremor, Balance, the total measure of the Parkinson’s Disease Questionnaire 39 (PDQ-39)^28^, MDS-Unified Parkinson’s disease rating (MDS-UPDRS) II and III^29^ and Quality-of-life. Here, the MDS-UPDRS Part II and Part III were combined as they both assess motor performance: Part II measures experiences of daily living and Part III the motor symptoms of PD. If both measures were provided, MDS-UPDRS III outcome measures were considered. The outcome measures in the category Quality-of-life consist of different measures contributing to the overall quality of one’s life, such as depression, activities of daily living or overall well-being.

#### Sub-level analysis

Four sub-level analyses were conducted, firstly to investigate whether the results from the primary measures demonstrated differences as levels of design ((R)CT and RT), and secondly as levels of outcome type (motor and cognitive). Furthermore, it was tested whether the results depended on cadence (high and low), and treatment duration (immediate vs. long-term effect). The ‘design’ level was applied to investigate whether (R)CT and RT studies differ in their effect sizes. The distinction between motor and cognitive outcome measures was applied to test whether either of the outcome types would gain a larger benefit from a bicycling intervention. For the sake of clarity, here the term ‘motor’ is being used, even though the term includes several types of physical parameters. Cadence and treatment duration were applied as sub-levels to test whether certain treatment-specific features would indicate better outcomes. Cadence was categorized as low when it was ≤60 RPM, and as high if it was ≥61 RPM. An effect was considered ‘immediate’ if it was performed only once, and ‘long-term’ if treatment sessions were more than one. All sub-level tests were performed on the primary outcomes.

#### Measures of heterogeneity

Due to the different designs and patient groups, as well as due to the various types of combined primary measures, it could be expected that there is clinical and statistical heterogeneity present in the pooled effect size^30^. Next to the 95% confidence intervals (CI), the *I²* is inspected as it gives a percentage estimate of the variability not caused by the sampling error and has an approximate rule of interpreting the results as small (25%), medium (50%) or large (75%) effects^31^. In addition, to investigate between-study heterogeneity, measures were taken to inspect the effect size contribution of individual studies. First, confidence interval (CI)-based outliers were detected using the meta package in R^22^. Second, to find out whether the results would change by not including some studies, a sensitivity analysis based on the Leave-One-Out method was chosen. This method reports several measures of between-study heterogeneity to test how much of the results would change if each study was left out at a time. Finally, studies that were outside the average confidence interval or contributed a particularly high influence were removed from the final pooling of the respective effect.

## Results

### Study characteristics

**Fig. 1:**
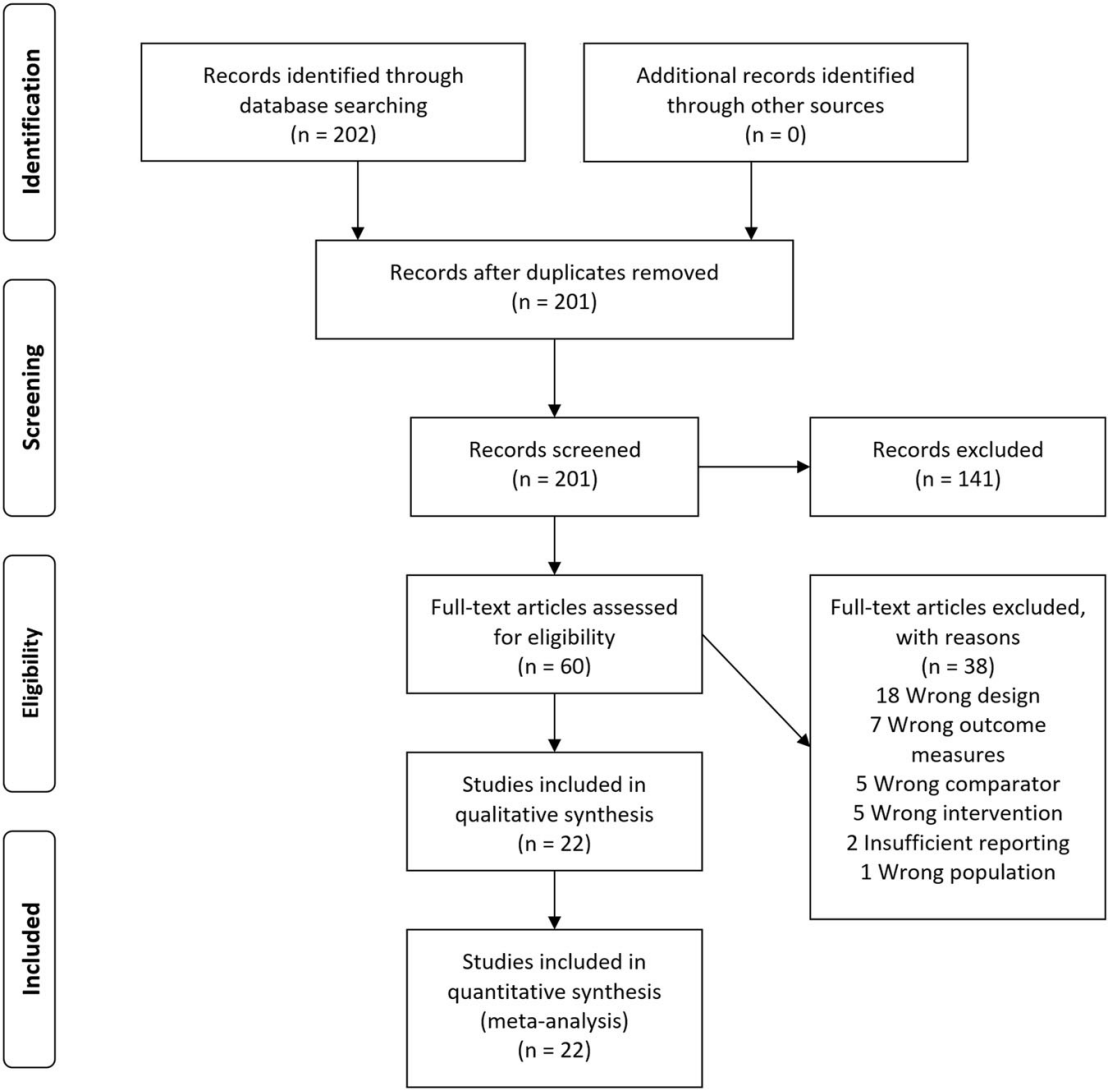
Literature search. Preferred Reporting Items for Systematic Reviews and Meta-analyses (PRISMA) flow diagram of the literature search.

The Preferred Reporting Items for Systematic Reviews and Meta-analyses (PRISMA) diagram represents the literature search (Fig. 1)^32^. See Table 1 below, and the Tables 5 and 6 in the supplementary material for further details on each study.

**Table 1:** Demographics and primary measures.

Study	Primary outcome	Outcome	C. group	F T	M T	F C	M C	Age		D. duration		H&Y		PD Medic. ON/OFF	Test time point	LEDD (mg)
								T	C	T	C	T	C			
Arcolin et al., 2016^48^	6-MWT	Motor	PD	7	9	7	6	67.8	68.7	4.7	6.5	2.3	2.3	ON	Within 2 h	T 887 (412) C 573 (366)
Demonceau et al., 2017^49^	Work load	Motor	PD^a^	4	12	5	10	65	63.3	5	5	1.5	1.5	ON	NA	T 390 (375–695) C 441 (210–750)
Ferraz et al., 2018^50^	6-MWT	Motor	PD	9	11	6	16	67	71	6	4	2.5	2.5	ON	NA	NA
Harper et al., 2019^51^	Executive Function	Cognition	PD^b^	20^d^		15^d^		65.05	64.87	NA	NA	NA	NA	ON	NA	T 532 (275) C 560 (557)
Qutubuddin et al., 2013^52^	MDS-UPDRS-III°	Motor	PD	13^d^		10^d^		68.2	68.2	7.2	7.2	NA	NA	ON	Within 3 h	NA
Ridgel et al., 2011a^53^	Tremor	Motor	PD^b^	7	13	3	9	62.8	64.6	5.2	6.5	2	1.6	OFF	8–12 h withholding	**
Tollár et al., 2019^35^	MDS-UPDRS II°	Motor	PD	14	11	11	13	70.6	67.7	7.5	7.3	2.4	2.4	ON	Within 1–2 h	T 786.4 (120.9) C 825.4 (126.6)
Means of the (R)CT-studies				130		111		66.6	66.9	5.9	6.1	2.14	2.06			
Chang, 2018^54^	MDS-UPDRS III	Motor	Rep.	4	9	4	9	59.7	59.7	6.44	6.44	1.96	1.96	OFF	12–24 h withholding	595 (246)
Corbett et al., 2013^55^	Hip extension	Motor	Rep.	4	13	4	13	64	64	8.7	8.7	1.8	1.8	Both	Abstain from morning med.	**
Duchesne et al., 2015^56^	TMT-A&B	Cognition	Rep.^c^	6	13	6	13	59	59	8.1	8.1	2	2	ON	NA	NA
Fiorelli et al., 2019^57^	TMT-B	Cognition	Rep.	6	6	6	6	66.5	66.5	5.7	5.7	NA	NA	ON	Within 1 h	NA
Hazamy et al., 2017^58^	Visual memory	Cognition	Rep.^c^	39^d^		39^d^		66.23	66.23	NA	NA	NA	NA	ON	NA	NA
McGough et al., 2016^59^	FTSTS	Motor	Rep.	16	25	16	25	62.7	62.7	5.4	5.4	NA	NA	ON	Within 2 h	NA
Nadeau et al., 2017^33^	Walking speed°	Motor	Rep.^c^	6	13	6	13	59	59	8.1	8.1	2.1	2.1	ON	NA	NA
Nadeau et al., 2019^60^	RT visual	Motor	Rep.^c^	6	13	6	13	59	59	8.1	8.1	2.1	2.1	NA	NA	NA
Peacock et al., 2014^61^	Muscle strength	Motor	Rep.^c^	13^d^		13^d^		67.6	67.6	NA	NA	NA	NA	ON	NA	NA
Ridgel et al., 2011b^62^	TMT-B	Cognition	Rep.	7	12	7	12	63	63	5.1	5.1	1.9	1.9	ON	Within 10 h	585 (561)
Ridgel et al., 2012^34^	Bradykinesia°	Motor	Rep.	6	4	6	4	64.5	64.5	6.5	6.5	1.8	1.8	Both	10–12 h withhold	751.6 (455.2)
Steib et al., 2018^63^	Balance	Motor	Rep.	4	13	4	13	64.4	64.4	NA	NA	2.1	2.1	ON	NA	NA
Tabak et al., 2013^64^	PDCRS	Cognition	Rep.	1	1	1	1	66.5	66.5	10.5	10.5	NA	NA	ON	NA	**
Uygur et al., 2015^65^	4SST	Motor	Rep.	1	9	1	9	64.6	64.6	4.1	4.1	1.95	1.95	ON	NA	NA
Uygur et al., 2017^66^	H&Y Scale	Motor	Rep.	4	10	4	10	62.4	62.4	3.3	3.3	2.54	2.54	ON	NA	885 (646)
Means of the RT-studies				264				63.3		6.7		2.03				
Overall means				505				64.3	64.4	6.4	6.5	2.06	2.04			

The table summarizes the main information of the 22 studies that were included into the meta-analysis.

Column *Primary outcome* shows the various measures defined as primary outcomes in the corresponding studies: 6-MWT (Six Minute Walk Test); MDS-UPDRS II-III (MDS-United Parkinson’s Disease Rating Scale II-III); TMT-A&B (Trail Making Test A & B); RT-visual (Visual reaction time); FTSTS (Five-Times-Sit-to-Stand); ROM (Range of Movement); PDCRS (Parkinson’s disease Cognitive Rating Scale); 4SST (Four Square Step Test). The degree symbol next to the measures indicates that the measure was moved due to large heterogeneity contribution, no symbol means that the specific outcome improved as a result of bicycling.

Column *Outcome* shows whether the variable was defined as Motor or Cognitive for the sub-level analysis.

Column *C. group* (Control group): PD = Control group was a different group of PD patients, Rep. = Control groups was the same group of PD patients as the intervention group. ^a^Study was pseudo-randomized. ^b^Study applied the same inclusion criteria for control and treatment groups but recruited the participants separately to each group. ^c^Study had a control group of healthy participants, but here the study was treated as a repeated design as the pre- and post-measures of the treatment groups were compared.

Columns *F/M T and F/M C* indicate the numbers of females and males per treatment and control group in each study. ^d^Study did not report the number of participants separately for each group. Column *Age T* and *C* provide the average age of the participants in treatment and control groups.

Column *D. duration* (Disease duration) *T* and *C* tell the average disease duration in years for the treatment and control groups. Column *H&Y T* and *C* indicate the average Hoehn & Yahr rating scale score of the participants in both groups before the treatment.

Column *PD medication ON/OFF* entails whether the participants were instructed to keep their medication as prescribed, or whether withdrawal was requested. Column *Testing time point* indicates how many hours prior to testing the last PD medication was taken.

Last column *LEDD* specifies the dose of the taken medication as measured in the Levodopa equivalent daily dose in milligrams. ^**^Dose reported but due to variability in the given range, LEDD cannot be calculated reliably.

*NA* means that the specific data were not available.

### Bicycling intervention characteristics

The treatment duration varied between 1 and 12 weeks with an average of 5.3 weeks, and a standard deviation of 4 weeks. Sessions per week varied between 1 and 5 sessions in a week. Out of the 22 included studies, three did not report the bicycling cadence (revolutions per minute, rpm), one reported it to be on a ‘comfortable’ level, while the remaining 18 reported the cadence. The cadence was binned in groups of 10 rpm, starting at 40–50 rpm and going up to 80–90 rpm. Nine out of the 18 studies reporting the cadence aimed at 70–80 rpm or at 80–90 rpm. An assisted bicycling intervention was reported by 41% of the studies, while 59% reported the intervention to have been non-assisted.

### Quality analysis

The *F*-test for the equality of the variances of the quality scores of both reviewers revealed that there was no significant variance *F*(1, 21) = 1.2, *p* = 0.34. Also, the following paired *t*-test confirmed that the quality scores did not vary significantly *t*(42) = 1.0, *p* = 0.33. The given scores ranged from 0.64 to 0.96, the best possible score being 1.0. The average score across all studies was 0.81 with a standard deviation of 0.08, and a median of 0.8. For further details on the quality analysis, see Supplementary Tables 1 and 2.

### Publication bias

**Fig. 2:**
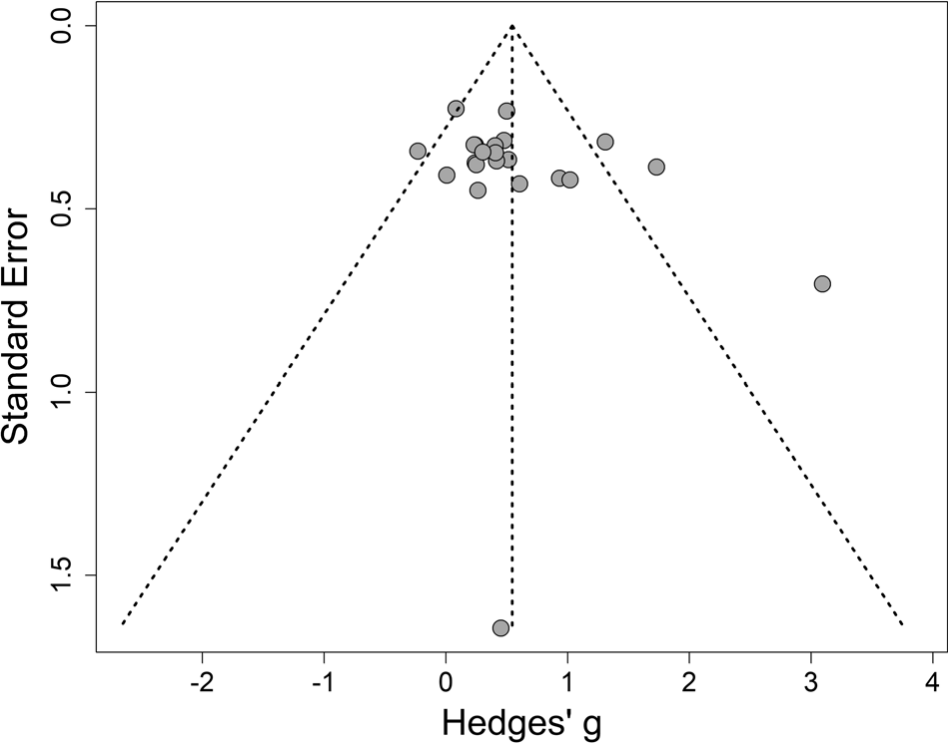
Funnel plot of the publication bias. Studies with a smaller sample size are expected to have higher standard error, and are therefore expected to be located at the lower end of the *y*-axis, while the studies with more participants are expected to show a lower standard error, and thus be at the upper end of the *y*-axis. SE = Standard error, Hedges’ *g* = The Hedges’ bias-corrected standardized mean difference which was used to calculate the effect size.

There is no significant publication bias present in the included studies as confirmed by Egger’s statistical test of the intercept on the funnel plot asymmetry (Intercept = 1.88; CI [−0.27, 4.04], *t* = 1.71], *p* = 0.10). In the case of publication bias, it could be expected that there are asymmetrically distributed small studies located low on the *y*-axis (high SE) and high on the *x*-axis (high ES) (Fig. 2).

### Primary measures

**Fig. 3:**
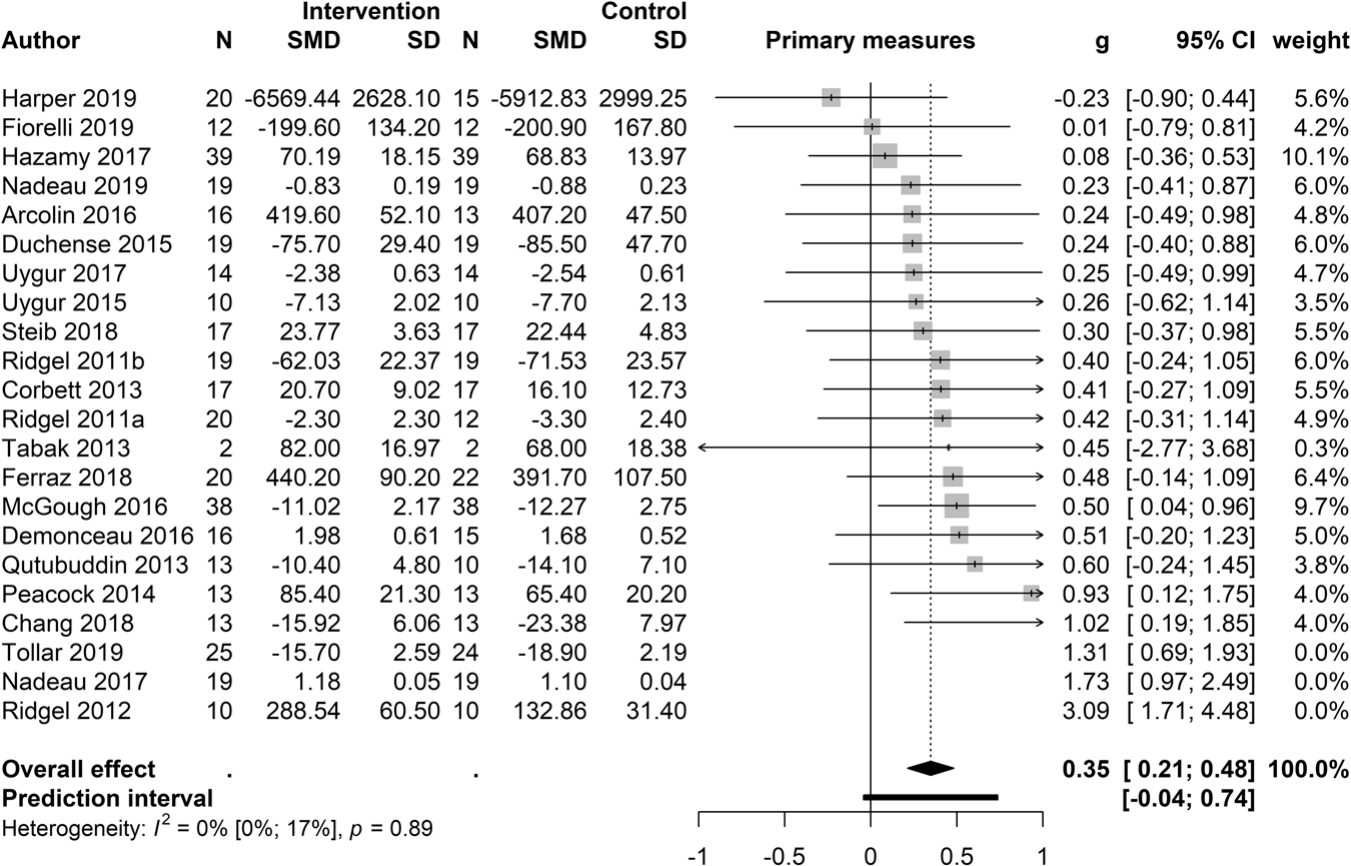
Primary outcome measures. The forest plot demonstrates how each study contributes to the effect size of the primary outcomes. The overall effect is marked with the diamond symbol on *x*-axis of the effect size scale. N = Number of patients; SMD = standardized mean difference; SD = standard deviation; *g* = the Hedges’ bias-corrected standardized mean difference, which was used to calculate the effect size; CI = 95% confidence interval; weight = the weight based on the inverse of the variance given to each study; *I*² = percentage of variability.

To test the overall efficacy of a bicycling intervention, the primary measures from all 22 studies were pooled together, and the pooled standardized mean difference (SMD) showed a significant effect (*k* 22; SMD 0.55; 95% CI [0.27, −0.82], *t* = 4.16, *p* < 0.001), yet also a medium-sized between-studies heterogeneity (*I²* =53.5%). Based on the outlier detection analysis, two studies (Nadeau et al., 2017^33^ and Ridgel et al., 2012^34^) were detected to be outside the CI due to large effect size influence and high heterogeneity. Furthermore, based on the influence analysis, an additional study (Tollár et al., 2019^35^) was marked as contributing to high heterogeneity and effect size. The latter was also marked by the Leave-One-Out-sensitivity method as a contributor to a high *I²*. Thus, three studies were removed from the final effect size pooling of the primary measures. After this procedure, the overall effect size is smaller, but there is also no substantial heterogeneity in the results (*k* 19; SMD 0.35; 95% CI [0.21, 0.48], *t* = 5.47, *p* < 0.001, *I²* =0,0%) (Fig. 3). The sub-level by the outcome type test (motor vs. cognitive) revealed a significant difference between the groups: *p* = 0.02, Motor 13; SMD 0.42; [0.27, 0.58] and Cognition 6; SMD 0.15; 95% CI [−0.11, 0.4]. When inspecting the results with respect to the treatment duration (immediate vs. long), the sub-level analysis revealed that a longer duration is more beneficial than a treatment taking place only once: *p* *=* 0.003, Long term 13; SMD 0.46; 95% CI [0.32, 0.6] and Immediate 6; SMD 0.13; 95% CI [−0.11, 0.36]. On average, the post-measures were taken 35 days after starting the intervention (standard deviation 29.8 days). The sub-level by ‘Design’ analysis revealed no significant difference between the groups: *p* *=* 0.5; (R)CT 7; SMD 0.29; 95% CI [0.00, 0.57] and RT 12; SMD 0.38; 95% CI [0.21, 0.55]. When cadence was applied as a sub-level, no significant difference between the groups of high and low cadence was detected: *p* *=* 0.8, Low 6; SMD 0.33; 95% CI [−0.03, 0.69] and High 8; SMD 0.37; 95% CI [0.12, 0.62]. Five studies were excluded as they did not report cadence.

### Secondary measures

**Fig. 4:**
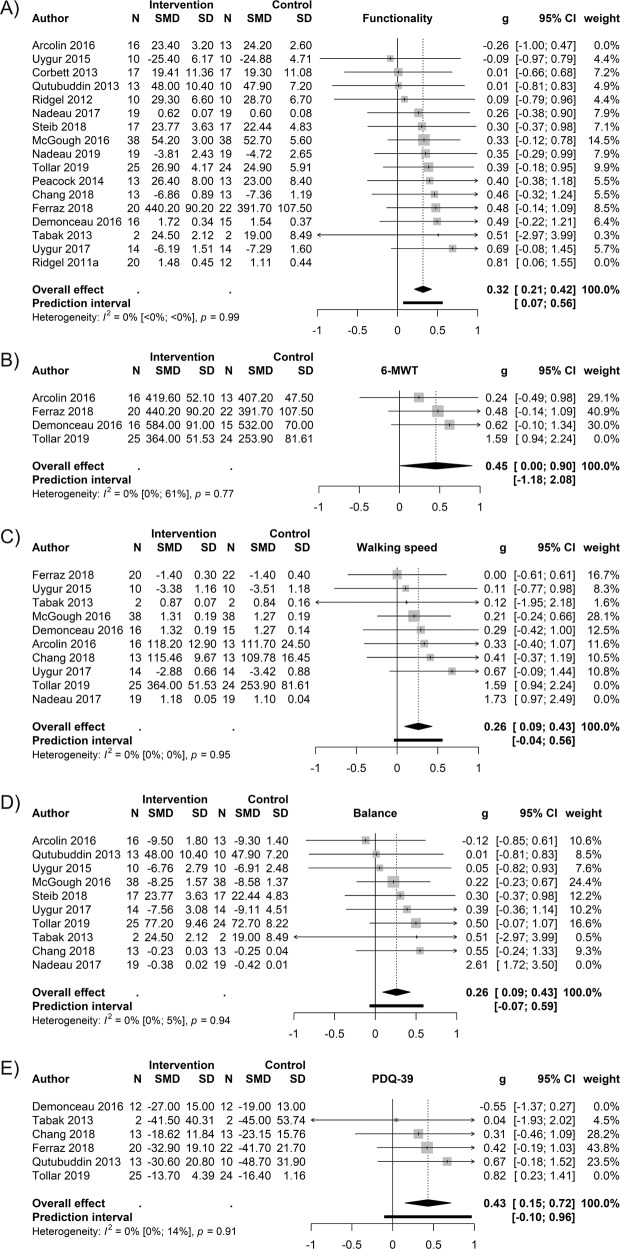
Secondary outcome measures. **A** Physical functionality. Arcolin et al. (2016)^48^ and Ridgel et al. (2011a)^53^ were removed based on the influence analysis. **B** Gait – 6-MWT. Tollár et al. (2019)^35^ was removed based on the influence analysis. **C** Gait – Speed. Tollár et al. (2019)^35^ and Nadeau et al. (2017)^33^ were removed based on the influence analysis. **D** Balance. Nadeau et al. (2017)^33^ was removed based on the CI-based outlier detection. **E** PDQ-39. Demonceau et al. (2017)^49^ and Tollár et al. (2019)^35^ were removed based on the influence analysis. Panels A–E enlist the forest plots of the secondary outcome measures indicating significant improvements as a result of bicycling. All plots demonstrate the results after removing possible outlier studies.

The forest plots A–E below (Fig. 4) depict the significant secondary outcome measures. The non-significant results of the secondary measures are presented in Supplementary Tables 3 and 4.

## Discussion

The present work highlights the beneficial effects of bicycling for patients suffering from Parkinson’s disease. Outcomes measuring motor parameters improved more from bicycling intervention when compared to the outcomes assessing cognitive performance. Also, when outcomes were grouped based on functionality across primary and secondary measures, a medium-sized improvement was demonstrated. We cannot address whether bicycling is best applied as a goal-oriented form of exercise, or whether it is beneficial also in the form of general physical activity. Nevertheless, it was indicated that interventions that are implemented more than once lead to better outcomes, thus demonstrating that longer-term regimens should be preferred over one-time sessions aiming at immediate effects when designing bicycling interventions. Overall, it is clear that bicycling improves motor outcomes in PD, and perhaps to a lesser extent, cognitive outcomes.

The effect size (SMD 0.43) based on the total score of PDQ-39 is an encouraging indication about the benefits of bicycling going beyond solely physical improvement, thus benefitting coping in daily life, and the self-rated overall quality-of-life. The PDQ-39 questionnaire assesses the overall self-reported quality-of-life of PD patients and it consists of 8 dimensions in a wide variety of measures related to difficulties in daily living (mobility, activities of daily living, emotional well-being, stigma, social support, cognition, communication and bodily discomfort)^36^. As the outcome measure ‘quality-of-life’ of this meta-analysis was not significant, it would be beneficial to address the PDQ-39 subscales in order to understand in which aspects of well-being and life-quality the improvement takes place. The difference in the PDQ-39 and the quality-of-life measure could possibly be that in the latter measure there is a too wide variety of aspects included, as they range from depression and disabilities to general daily living and well-being. For further details on the measures included to the outcome quality-of-life please see Supplementary Table 6.

There is previous evidence that moderate- to high-intensity physical exercise is well tolerated by PD patients, leading to better outcomes than low-intensity training^37,38^. In this meta-analysis it was addressed whether the primary outcome measures indicated a difference in the effect size depending on whether the cadence was high or low, but no difference in the outcomes was found. However, not all studies reported the cadence, and cadence alone is not a sufficient measure of training intensity. Other measures of intensity, such as heart rate and rate of perceived exertion were reported rather variably, either not at all, in different units, or they were merely monitored, thus drawing further conclusion about the role of intensity is not feasible based on the data at hand. Despite some of the here-included studies already systematically varying intensity and other exercise-programme-related parameters, more comparative studies are needed to better understand the customizability of bicycling. This is an important notion, as bicycling has the potential of catering to both high- and low-intensity exercise while allowing the customization of the intensity of skeletomuscular activation, and overall mobility by varying the ratio of cadence and resistance.

Recent meta-analyses have reported that FOG can benefit from physiotherapy and from physical exercise in general^2,39^. Due to lack of FOG being an outcome measure in the studies included here, this meta-analysis cannot provide any information about the influence of bicycling on FOG. Thus, it would be crucial to further investigate the possible benefits of bicycling in particular on patients suffering from freezing for an enhanced customization of a possible bicycling intervention.

Furthermore, many exercise protocols in the reviewed studies implemented a recumbent or a stationary bicycle, meaning that the patients’ ability to balance was not being as challenged as it would be on a regular bicycle. Nevertheless, the results demonstrate that balance improved as an outcome of the applied interventions. Thus, when developing technically more advanced forms of bicycling exercises, it might be worth aiming at regimens where balancing is similarly challenged as on a regular bicycle, as it could be expected to benefit the balancing outcome even more. Moreover, it has been reported that balance training reduces fear of falling^40^, which is known to be one of the most disabling symptoms in PD. Thus, an improved balance as an outcome of bicycling could be expected to enhance other life-limiting challenges of PD patients as well^41,42^.

Patients’ own motivation, and possible barriers of exercising are a major factor in the success of physical exercise and overall activity^43^. Importance of considering safety and preference features have been suggested to be a decisive factor, in whether clinician promotes treadmill or cycling to a patient^44^. In the included studies, no conclusions can be drawn about the subjective ratings towards the exercise itself. Thus, for a successful clinical practice there clearly is a need to assess patients’ own judgement of the exercise programme, as well as the overall suitability in terms of practical implementation into one’s own daily life. Furthermore, for future studies it would be beneficial to assess for any differences in targeting exercise to early, middle or later disease stages^39,44^.

The main concern when including NRCT studies is that the baseline measurements of the different groups are not equal due to a lack of randomization in the treatment allocation, or due to differences in experimental designs thus possibly leading to biased results^45^. To observe and minimize any possible bias, several methodological precautions were taken. Firstly, a thorough and versatile assessment of quality designed to include also NRCT studies was applied. Furthermore, random-effects model was chosen over a fixed-effects model to counterbalance the possibly heterogeneous patient population. Also, various measures of heterogeneity and sensitivity were applied to point at any studies contributing to a large heterogeneity. Lastly, the primary outcomes were inspected on the sub levels of study designs to test whether the design led to differences in the found effect sizes.

The quality assessment criteria, QualSyst, was applied to evaluate the quality of reporting, the internal validity of the included studies, and the certainty of the findings of individual studies. Since the *F*-test, the subsequent *t*-test of the assessment done by the two reviewers were non-significant it can be concluded that the reviewers agreed sufficiently well on the outcomes of the assessed items. Furthermore, on average the reviewed studies scored good ratings. The between-study heterogeneity assessment and the sensitivity analysis, with the subsequent removal of identified studies and their respective outcome measures from the pooling of the effect-size are considered as an indication of the certainty of the results. Conclusively, the certainty of the overall results of the presented studies would mainly benefit from enhancing the design, favouring RCT would lead to an increased overall controllability. Furthermore, increasing sample size and the unification of certain measures as well as intervention protocols could increase the certainty and overall quality of the findings.

The present work demonstrates that bicycling can lead to versatile improvements, yet it also seems that the effects of bicycling are rather specific, and when it comes to a more detailed understanding, or prescribing physical exercise regimen based on personalized needs and preferences, the current knowledge remains scattered. More studies are needed to directly address the potential benefit of bicycling on the most common, functionally and psychologically disabling symptoms such as falling and FOG^41,46^. Overall, in order to understand in which situations bicycling is best applied, over other forms of exercise, more scrutiny on the reporting and controlling of the intervention, and the outcomes is needed. This would be particularly important in order to define the optimal intensity and cadence of bicycling exercise, as well as to recognize the optimal stage of disease progression at which the training could be most beneficial. As the currently available pharmaceutical medication for PD only treats the symptoms, at best improving the daily coping of the patients while not terminating the disease progression^47^, developing well-targeted adjuvant forms of physical exercise is crucial.

## Conclusion

Taken together, this review provides evidence that bicycling is a versatile form of physical exercise for PD patients. Considering the clinical relevance of the findings, the results support the application of bicycling, in particular to improve gait-related parameters of balance, walking speed and overall walking capacity. Furthermore, based on the outcome measure PDQ-39, the benefits of bicycling go beyond physical improvement, resulting in an increased quality of daily living. In addition, the results indicate that the effects of bicycling are based on longer-term exercise rather than on immediate effects of single sessions. Therefore, bicycling is a meaningful way to improve the lives of patients suffering from Parkinson’s disease.

## Supplementary information

Supplementary information.

## Data Availability

All data analysed in this work are based on already published data, which is referenced and, where applicable, presented in the main manuscript and supplementary material. A pre-registration of the meta-analysis can be found in the International Prospective Register of Systematic Reviews (PROSPERO) with the number CRD42019137386.
