# Supplementary material for: Parkinson’s disease patients benefit from bicycling - a systematic review and meta-analysis
Source: NPJ Parkinsons Dis. 2021 Sep 24;7:86. doi: 10.1038/s41531-021-00222-6 (PMC8463550; doi:10.1038/s41531-021-00222-6)
Supplement: Supplementary file 1 — Supplementary information. [file 41531_2021_222_MOESM1_ESM.pdf]

Supplementary Material

Supplementary Material: Table 1 (page 2). Quality Assessment Criteria

Supplementary Material: Table 2 (page 3). Quality Assessment Score

Supplementary Material: Table 3 (page 4). Non-significant secondary measure results

Supplementary Material: Table 4 (page 4). Non-significant secondary measure studies

Supplementary Material: Table 5 (page 5). Intervention details

Supplementary Material: Table 6 (page 9). Study details and measures

Table 1

Quality Assessment Criteria

**1.**	Question / objective sufficiently described?
**2.**	Study design evident and appropriate?
**3.**	Method of subject/comparison group selection or source of information/input variables described and appropriate?
**4.**	Subject (and comparison group, if applicable) characteristics sufficiently described?
**5.**	If interventional and random allocation was possible, was it described?
**6.**	If interventional and blinding of investigators was possible, was it reported?
**7.**	If interventional and blinding of subjects was possible, was it reported?
**8.**	Outcome and (if applicable) exposure measure(s) well defined and robust to measurement / misclassification bias? means of assessment reported?
**9.**	Sample size appropriate?
**10.**	Analytic methods described/justified and appropriate?
**11.**	Some estimate of variance is reported for the main results?
**12.**	Controlled for confounding?
**13.**	Results reported in sufficient detail?
**14.**	Conclusions supported by the results?

Table 1. Quality assessment criteria for quantitative studies. [1]

Each item could be answered with *Yes, No, Partially, or Not applicable*. The overall score is a sum of the items, each answer having a certain weight.

Table 2

Overall quality score

Author	Reviewer I	Reviewer II	Mean
Arcolin et al., 2016[2]	0.93	0.96	0.95
Chang et al., 2018[3]	0.86	0.86	0.86
Corbett et al., 2013[4]	0.86	0.69	0.78
Demonceau et al., 2016[5]	0.73	0.85	0.79
Duchesne et al., 2015[6]	0.88	0.95	0.92
Ferraz et al., 2018[7]	0.92	0.88	0.9
Fiorelli et al., 2019[8]	0.81	0.83	0.82
Harper et al., 2019[9]	0.69	0.86	0.76
Hazamy et al., 2017[10]	0.79	0.69	0.74
McGough et al., 2016[11]	0.77	0.77	0.77
Nadeau et al., 2017[12]	0.71	0.91	0.81
Nadeua et al., 2018[13]	0.75	0.95	0.85
Peacock et al., 2014[14]	0.79	0.82	0.81
Qutubuddin et al., 2013[15]	0.88	0.81	0.85
Ridgel et al., 2011a[16]	0.73	0.73	0.73
Ridgel et al., 2011b[17]	0.79	0.83	0.81
Ridgel et al., 2012[18]	0.71	0.73	0.72
Steib et al., 2018[19]	0.71	0.82	0.77
Tabak et al., 2013[20]	0.7	0.72	0.71
Tollár et al., 2019[21]	0.96	0.96	0.96
Uygur et al., 2015[22]	0.79	0.75	0.77
Uygur et al., 2017[23]	0.65	0.64	0.65

Table 2: Scores from both reviewers and an average of the two means.

Table 3

Non-significant secondary measure results

Measure	N	SMD	95% CI	*t*	*p*	I^2 %	Removed studies
**Cadence**	6	0.6047	[0.00, 1.21]	2.59	0.0491	60.5	Nadeau 2017
	5	0.4270	[-0.09, 0.94]	2.29	0.0838	24.3	
**Step length**	5	0.1548	[-0.08, 0.39]	1.85	0.1379	0.00	
**Bradykinesia**	4	1.3	[-0.45, 3.04]	2.37	0.0985	73.0	
**Tremor**	4	0.18	[-0.14, 0.51]	1.77	0.1746	0.00	
**UPDRS II-III**	6	0.55	[-0.06, 1.17]	2.31	0.0689	63.3	Tollár 2019
	5	0.37	[-0.23, 0.98]	1.7	0.16	37.3	
**Quality-of-life**	8	0.23	[-0.14, 0.60]	1.46	0.1883	25.7	

Table 3: The table shows the non-significant effect sizes of the secondary outcome measures.

Measure = Outcome measure, N = Number of included studies, SMD = Standardized mean difference, 95% CI = 95% confidence interval, t = t-statistics, p = probability of the detected effect size, I^2 % = A percentage estimate of the variability not caused by the sampling error.

The column *Removed studies* indicates the studies that were detected as outliers based on the sensitivity analysis contributing with large effect size and / or heterogeneity, and thus removed from the final pooling. In the table, the effect size measure on the row below the removed study reports the final effect size of the corresponding measure, without the removed study.

Table 4

Non-significant secondary measure studies

Measure	Studies						
**Gait cadence**	Nadeau 2017	Chang 2018	McGough 2016	Uygur 2017	Arcolin 2016	Demonceau 2016	
**Step length**	Nadeau 2017	Chang 2018	McGough 2016	Arcolin 2016	Demonceau 2016		
**Bradykinesia**	Uygur 2017	Chang 2018	Ridgel 2011a	Ridgel 2012			
**Tremor**	Nadeau 2019	Chang 2018	Ridgel 2011a	Ridgel 2012			
**UPDRS II - III**	Tollár 2019	Nadeau 2019	Chang 2018	Uygur 2017	Arcolin 2016	Qutubuddin 2013	
**Quality of life**	Tollár 2019	Nadeau 2019	Uygur 2017	Qutubuddin 2013	Demonceau 2016	Ferraz 2018	Harper 2019	Tabak 2013

Table 4: The Table contains the studies that were included into the initial analysis of the above presented non-significant secondary measures.

Table 5

Intervention details

First author	Intervention	Design	Recruitment	Setting	Treatment provider	Att rition	Session duration (min)	Sessions / week	Overall duration (days)	Assisted	RPM	RPE	Heart rate
**Arcolin 2016**	Ergometer	RCT Pilot	Neurorehab. Center	Rehab. Center Laboratory	NA	0	30	5	21	No	60	11 to 14	NA
**Demonceau 2016**	Ergometer	RCT *	Movm. Dis. Clinic	Hospital Laboratory	Physiotherapist & Students	6	75	2.5	84	No	NA	12.3	Monit.
**Ferraz 2018**	Ergometer	RCT Pilot	Outpatient Clinic	Outpatient Clinic	Physiotherapist	10	50	3	56	No	NA	15	50 - 75 %
**Harper 2019**	Ergometer	RCT *	NA	Laboratory	NA	2	40	2	1	Yes	78	11.2	88.4
**Qutubuddin 2013**	Bicycle	RCT	Hospital advert	Medical Center	Neuropsychologist, assistant evaluators	14	30	2	56	Yes	NA	NA	NA
**Ridgel 2011a**	Bicycle	RCT	Support group, Neurology clinics	Laboratory	Laboratory assisstant	0	30	1	21	Yes	70	6 to 8	73
**Tollár 2019**	Ergometer	RCT	Database	Outpatient Physiotherapy Clinic	Physical therapists	0	60	5	35	No	NA	13.6	119.5 bpm
**Chang 2018**	Bicycle	RD	NA	NA	Trainer	0	35	2	56	No	40	Monit.	50 - 55 %
**Corbett 2013**	Recumbent Bicycle	RD	Support group, Neurology clinics	NA	NA	1	30	1	1	Yes	80	NA	60 - 70 %
**Duchesne 2015**	Recumbent bicycle	RD, HC	NA	NA	Kinesiologist	0	30	3	21	No	60	Monit.	60 - 80 %

**Fiorelli 2019**	Bicycle	RD	Hospital PD group	NA	NA	2	30	1	1	No	50-60	15 to 17	NA
**Hazamy 2017**	Bicycle	RD, HC	Center for Mov. Dis., Database, Word of mouth	NA	NA	0	5	1	1	No	Comf.	NA	Monit.
**McGough 2016**	Tandem bicycle	RD	Newsletter, PD registery, Activity groups	NA	Class instructor & Tandem partners	3	60	3	70	Yes	85	Monit.	Monit.
**Nadeau 2017**	Bicycle	RD, HC	NA	NA	Kinesiologist	3	30	3	84	No	60	Monit.	Max 80 %
**Nadeau 2019**	Bicycle	RD, HC	NA	NA	Kinesiologist	3	40	3	84	No	60	Monit.	NA
**Peacock 2014**	Ergometer	RD, HC	Local community, Support group	Exercise physiology laboratory	Certified personal trainer	2	30	3	56	Yes	80	11 to 16	110 - 160 bpm
**Ridgel 2011b**	Ergometer	RD	NA	NA	NA	0	40	1	21	Yes	80	6 to 8	73 bpm
**Ridgel 2012**	Ergometer	RD Pilot	NA	NA	Laboratory assistant	0	40	1	1	Yes	80	13.3	98 bpm
**Steib 2018**	Ergometer	RD	NA	NA	Exercise therapist	0	30	1	1	No	70	6 to 20	60 - 70 %
**Tabak 2013**	Bicycle	Case-series	Movm. Dis. Clinic, Support groups, PD organisations	NA	Researchers	0	40	3	56	No	69	Monit.	97 bpm

**Uygur 2015**	Recumbent bicycle	RD	NA	NA	NA	0	30	1	1	Yes	99	NA	Monit.
**Uygur 2017**	Recumbent bicycle	RD	Support group	NA	Experienced trainer	0	30	2	42	Yes	NA	13.7	Monit.

Table 5: Intervention details

The column *Intervention* tells which type of bicycle was used to deliver the intervention.

The column *design tells the design of each study: RCT = randomized control trial, RCT* = pseudo randomized or study applied the same inclusion and exclusion criteria to both groups but recruited them separately, RD = repeated design, HC = healthy controls.*

The column *assisted* tells whether the bicycling intervention was assisted, e.g. by an ergometer motor.

***RPM* = Rounds per minute, cadence.**

***RPE* = Rate of perceived exertion, reports the subjective rating of the experienced effort during the exercise based on the Borg Scale.**

The column *Heart Rate* shows whether the heart rate was only monitored during the test, or if it was measured, it is reported either as a percentage of maximum capacity (%), or as an average of beats per minute (bpm).

***NA* means that no data was available**

Table 6

Study details and measures

Author	Year	Journal	Country	Used test, scale or questionnaire	Outcome measure
**Arcolin**	2016	Restor Neurol Neurosci	Italy	6-MWT, W. capacity	PM (Motor), Physical functioning, 6-MWT
				Gait speed (cm / s)	Gait speed
				Step length (cm)	Step length
				Cadence (Steps / min)	Gait cadence
				TUG-test	Balance
				Mini-BesTest	Physical functioning
				MDS-UPDRS motor	MDS-UPDRS II - III
**Demonceau**	2016	Eur J Phys Rehabil Med	Belgium	Peak torq. knee extension	Physical functioning
				Peak work load (Strength)	PM (Motor)
				Speed (Time to cover 30 meters)	Gait speed
				Stride length (Speed / Cadence)	Step length
				Cadence (Trunk accelerometer)	Gait cadence
				6-MWT, W. capacity	6-MWT
				PDQ-39 Total	PDQ-39, Quality of life
**Ferraz**	2018	Arch Phys Med Rehabil	Brazil	6-MWT, W. capacity	PM (Motor), Physical functioning, 6-MWT
				10-Meter walk test	Gait speed
				PDQ-39 Total	PDQ-39
				WHODAS 2.0 (Disability)	Quality of life
**Harper**	2019	Int J Environ Res Public Health	USA	WebNeuro (Executive function)	PM (Cognition)
				BDI-II (Depression, Wellbeing)	Quality of life
**Qutubuddin**		Rehabil Res Pract	USA	MDS-UPDRS-III	PM, MDS-UPDRS II - III
				BBS	Physical functioning, Balance
				PDQ-39 total	PDQ-39, Quality of life
**Ridgel**	2011a	Phys Sportsmed	USA	Accelerometer, Gyroscope (Tremor)	PM (Motor), Tremor
				Accelerometer, Gyroscope, Hand grasp (Bradykinesia)	Bradykinesia
				Bradykinesia pronation / supination	Physical functioning
**Tollar**	2019	Gerontology	Hungary	MDS-UPDRS M-EDL (II)	PM (Motor), MDS-UPDRS II - III

				PDQ-39 Mobilitiy sub	PDQ-39
				EQ-5D-Questionnaire	Quality of life
				BBS, Balance, falling	Physical functioning
				BESTest, Balance control	Balance
				6-MWT, W. capacity	Gait speed
**Chang**	2018	J Clin Neurol	Taiwan	MDS-UPDRS III, Motor	PM (Motor), MDS-UPDRS II - III
				MDS-UPDRS III, Tremor sub	Tremor
				MDS-UPDRS III, Akinesia sub	Bradykinesia
				TUG-Test (Balance)	Physical functioning
				SE-ADL (Percentage of independece)	Quality of life
				PDQ-39 total	PDQ-39
				Gait speed (cm / s)	Gait speed
				Step length (cm)	Step length
				Step time (s)	Gait cadence
				Double limb support time (s)	Balance
**Corbett**	2013	NeuroRehabilitation	USA	Range of moevement, Hip, Mobility	PM (Motor)
				Gait, Hip extension, Mobility	Physical functioning
**Duchesne**	2015	Brain Cogn	Canada	TMT A&B	PM (Motor)
**Fiorelli**	2019	J Phys Act Health	Brazil	Cognition, Execution, Executive, motor	PM (Cognition)
**Hazamy**	2017	Brain Cogn	USA	Visual memory, execution, reaction time	PM (Cognition)
**McGough**	2016	J Neurol Phys Ther	USA	BBS, Balance	Physical functioning
				FTSTS, Lower extremity, Balance	PM (Motor)
				TUG, Balance	Balance
				Speed (m / s)	Speed
				Cadence (steps / min)	Gait cadence
				Stride length (m)	Step length
**Nadeau**	2017	Front Hum Neurosci	Canada	Gait speed (m / s)	PM (Motor), Speed
				Cadence steps (steps / min)	Gait cadence
				Step length (m)	Physical functioning, Step length
				Single support time (s), Balance	Balance

**Nadeau**	2019	Front Hum Neurosci	USA	MDS-UPDRS III sub, tremor	Tremor
				MDS-UPDRS III sub, rigidity	Physical functioning
				MDS-UPDRS III total subscore	MDS-UPDRS II - III
				Visuomotor (reaction time)	PM (Cognition)
**Peacock**	2014	Aging Clin Exp Res	USA	Sit and reach, Flexibility	Physical functioning
				Leg press, Muscle strength	PM (Motor)
**Ridgel**	2011b	J Aging Phys Act	USA	TMT-B	PM (Cognition)
**Ridgel**	2012	Arch Phys Med Rehabil	USA	Tremor score, 3D Gyroscope	Tremor
				Bradykinesia, 3D Gyroscope	PM (Motor), Bradykinesia
**Steib**	2018	Front Aging Neurosci	Germany	Time in balance	PM (Motor), Physical functioning, Balance
**Tabak**	2013	J Neurol Phys Ther	USA	PDCRS	PM (Cognition)
				PDQ-39 Total	PDQ-39, Quality of life
				Gait speed (m / s)	Speed
				FGA, Balance, Stability	Physical functioning, Balance
**Uygur**	2015	Physiother Theory Pract	USA	4SST, Balance	PM (Motor)
				TUG	Balance
				10MW	Speed
				9-HPT	Physical functioning
**Uygur**	2017	Physiother Theory Pract	USA	MDS-UPDRS III	MDS-UPDRS II - III
				MDS-UPDRS Bradykinesia	Bradykinesia
				H&Y Scale	PM (Motor)
				SF36	Quality of life
				10MW	Speed
				Steps	Gait cadence
				TUG	Physical functioning
				4SST, Balance, Stability	Balance

Table 6: Study details and measures

Column *Journal* = The journal name as abbreviated in the catalogue of the National Library of Medicine (NLM)

Column *Used test, scale or questionnaire* entails the abbreviations of the measures that were applied in the corresponding studies: 6-MWT = 6 Minute walk test; 10MWT = 10 Meter walk test; PDQ-39 = Parkinson‘s Disease Questionnaire; TUG-Test = Time Up and Go Test; Mini-BesTest = Mini Balance Evaluation Systems Test; WHODAS 2.0 = World Health Organisation Disability Assessment Schedule; TMT-B = Trail Making Test – B; WebNeuro = World-Wide-Web based neurocognitive assessment battery; BDI-II = Beck Depression Inventory II; MDS-UPDRS III = Movement Disorder Society-Sponsored; Revision of the Unified Parkinson's Disease Rating Scale III, Motor; BBS = Berg Balance Scale; MDS-UPDRS M-EDL II = Movement Disorder Society-Sponsored Revision of the Unified Parkinson's Disease Rating Scale, Motor Experience of Daily Living II; EQ-5D Euro Quality of Life 5-Dimensions; SE-ADL = Schwab and England Activities of Daily Living; FTSTS = Five Time Sit to Stand Test; PDCRS = Parkinson's Disease Cognitive Rating Scale; FGA = Functional Gait Assessment; 4SST = 4 Square Step Test; 9HPT = Nine Hole Peg Test; H&Y Scale = Hoehn and Yahr Scale; SF-36 = Short Form-36 Health Survey.

The column *Outcome measure* indicates how the corresponding measures was categorized for the analysis: PM (Motor) = Primary measure Motor; PM (Cognition) = Primary measure Cognition; 6-MWT = 6 Minute walk test; MDS-UPDRS III = Movement Disorder Society-Sponsored; Revision of the Unified Parkinson's Disease Rating Scale III; MDS-UPDRS M-EDL II = Movement Disorder Society-Sponsored Revision of the Unified Parkinson's Disease Rating Scale, Motor Experience of Daily Living II.

References

1. Kmet, L. M., Lee, R. C. & Cook, L. S. Standard quality assessment criteria for. *HTA Initiat.* **13**, (2004).
2. Arcolin, I. *et al.* Intensive cycle ergometer training improves gait speed and endurance in patients with Parkinson's disease: A comparison with treadmill training. *Restor. Neurol. Neurosci.* **34**, 125–138 (2016).
3. Chang, H.-C. An 8-Week Low-Intensity Progressive Cycling Training. **14**, 225–233 (2018).
4. Corbett, D. B., Peer, K. S. & Ridgel, A. L. Biomechanical muscle stimulation and active-assisted cycling improves active range of motion in individuals with Parkinson's disease. *NeuroRehabilitation* **33**, 313–322 (2013).
5. Demonceau, M. *et al.* Effects of 12 weeks of aerobic or strength training in addition to standard care in Parkinson ' s disease : a controlled study . EUROPEAN JOURNAL OF PHYSICAL AND REHABILITATION Subscription : Information about subscribing to Minerva Medica journals is onl. (2016).
6. Duchesne, C. *et al.* Enhancing both motor and cognitive functioning in Parkinson's disease: Aerobic exercise as a rehabilitative intervention. *Brain Cogn.* **99**, 68–77 (2015).
7. Ferraz, D. D. *et al.* The Effects of Functional Training, Bicycle Exercise, and Exergaming on Walking Capacity of Elderly Patients With Parkinson Disease: A Pilot Randomized Controlled Single-blinded Trial. *Arch. Phys. Med. Rehabil.* **99**, 826–833 (2018).
8. Fiorelli, C. M. *et al.* Differential acute effect of high-intensity interval or continuous moderate exercise on cognition in individuals with Parkinson's disease. *J. Phys. Act. Heal.* **16**, 157–164 (2019).
9. Harper, S. A., Dowdell, B. T., Kim, J. H., Pollock, B. S. & Ridgel, A. L. Non-motor symptoms after one week of high cadence cycling in Parkinson's disease. *Int. J. Environ. Res. Public Health* **16**, (2019).
10. Hazamy, A. A. *et al.* Improved cognition while cycling in Parkinson's disease patients and healthy adults. *Brain Cogn.* **113**, 23–31 (2017).
11. McGough, E. L. *et al.* A tandem cycling program: Feasibility and physical performance outcomes in people with Parkinson disease. *J. Neurol. Phys. Ther.* **40**, 223–229 (2016).
12. Nadeau, A. *et al.* A 12-week cycling training regimen improves gait and executive functions concomitantly in people with parkinson's

disease. *Front. Hum. Neurosci.* **10**, 1–10 (2017).

13. Nadeau, A. *et al.* A 12-Week Cycling Training Regimen Improves Upper Limb Functions in People With Parkinson's Disease. *Front. Hum. Neurosci.* **12**, 1–11 (2019).

14. Peacock, C. A. *et al.* Introducing a multifaceted exercise intervention particular to older adults diagnosed with parkinson's disease: A preliminary study. *Aging Clin. Exp. Res.* **26**, 403–409 (2014).

15. Qutubuddin, A. *et al.* Parkinson's Disease and Forced Exercise: A Preliminary Study. *Rehabil. Res. Pract.* **2013**, 1–5 (2013).

16. Ridgel, A. L., Muller, M. D., Kim, C. H., Fickes, E. J. & Mera, T. O. Acute effects of passive leg cycling on upper extremity tremor and bradykinesia in Parkinson's disease. *Phys. Sportsmed.* **39**, 83–93 (2011).

17. Ridgel, A. L., Kim, C. H., Fickes, E. J., Muller, M. D. & Alberts, J. L. Changes in executive function after acute bouts of passive cycling in Parkinson's disease. *J. Aging Phys. Act.* **19**, 87–98 (2011).

18. Ridgel, A. L., Peacock, C. A., Fickes, E. J. & Kim, C. H. Active-assisted cycling improves tremor and bradykinesia in Parkinson's disease. *Arch. Phys. Med. Rehabil.* **93**, 2049–2054 (2012).

19. Steib, S. *et al.* A Single Bout of Aerobic Exercise Improves Motor Skill Consolidation in Parkinson's Disease. *Front. Aging Neurosci.* **10**, (2018).

20. Tabak, R., Aquije, G. & Fisher, B. E. Aerobic exercise to improve executive function in Parkinson disease: A case series. *J. Neurol. Phys. Ther.* **37**, 58–64 (2013).

21. Tollar, J., Nagy, F. & Hortobágyi, T. Vastly Different Exercise Programs Similarly Improve Parkinsonian Symptoms: A Randomized Clinical Trial. *Gerontology* **65**, 120–127 (2019).

22. Uygur, M. *et al.* Immediate effects of high-speed cycling intervals on bradykinesia in Parkinson's disease. *Physiother. Theory Pract.* **31**, 77–82 (2015).

23. Uygur, M., Bellumori, M. & Knight, C. A. Effects of a low-resistance, interval bicycling intervention in Parkinson's Disease. *Physiother. Theory Pract.* **33**, 897–904 (2017).
